# Mechanosensing at the Nuclear Envelope by Nuclear Pore Complex Stretch Activation and Its Effect in Physiology and Pathology

**DOI:** 10.3389/fphys.2019.00896

**Published:** 2019-07-12

**Authors:** F. Donnaloja, E. Jacchetti, M. Soncini, M. T. Raimondi

**Affiliations:** ^1^Department of Chemistry, Materials and Chemical Engineering “Giulio Natta,” Politecnico di Milano, Milan, Italy; ^2^Department of Electronics, Information and Bioengineering, Politecnico di Milano, Milan, Italy

**Keywords:** nuclear pore complex, nuclear basket, mechanotransduction, stretch activation, pathologies

## Abstract

Cell fate is correlated to mechanotransduction, in which forces transmitted by the cytoskeleton filaments alter the nuclear shape, affecting transcription factor import/export, cells transcription activity and chromatin distribution. There is in fact evidence that stem cells cultured in 3D environments mimicking the native niche are able to maintain their stemness or modulate their cellular function. However, the molecular and biophysical mechanisms underlying cellular mechanosensing are still largely unclear. The propagation of mechanical stimuli via a direct pathway from cell membrane integrins to SUN proteins residing in the nuclear envelop has been demonstrated, but we suggest that the cells’ fate is mainly affected by the force distribution at the nuclear envelope level, where the SUN protein transmits the stimuli via its mechanical connection to several cell structures such as chromatin, lamina and the nuclear pore complex (NPC). In this review, we analyze the NPC structure and organization, which have not as yet been fully investigated, and its plausible involvement in cell fate. NPC is a multiprotein complex that spans the nuclear envelope, and is involved in several key cellular processes such as bidirectional nucleocytoplasmic exchange, cell cycle regulation, kinetochore organization, and regulation of gene expression. As several connections between the NPC and the nuclear envelope, chromatin and other transmembrane proteins have been identified, it is reasonable to suppose that nuclear deformations can alter the NPC structure. We provide evidence that the transmission of mechanical forces may significantly affects the basket conformation via the Nup153-SUN1 connection, both altering the passage of molecules through it and influencing the state of chromatin packing. Finally, we review the known correlations between a pathological NPC structure and diseases such as cancer, autoimmune disease, aging and laminopathies.

## Introduction

Cells respond to extracellular environment changes via their mechanosensitive elements, from the adhesion complexes to the nucleus itself (Ribbeck and Görlich, 2002; Wang et al., 2009; Kirby and Lammerding, 2018). The cells anchor the extracellular matrix due to the integrins and form focal adhesions as cluster of several proteins mainly composed of kinases, talin and vinculin. If activated, they transmit external mechanical stimuli to the actin fibers and other cytoskeleton filaments, which transfer the force to the LINC complex (LInker of Nucleoskeleton and Cytoskeleton) with a transmission rate of about 2 μs/50 μm (Jean et al., 2004; Wang et al., 2009; Jahed et al., 2014; Nava et al., 2014). The LINC complex is fundamental for the transmission of mechanotransduction events and completes the physical link between the cytoskeleton and the nuclear structures by enabling the entire cell to act as a mechanically coupled system. The LINC alteration results in disturbed intracellular force transmission (Lombardi et al., 2011). Once the force reaches this complex, it splits into different pathways according to the LINC connections at the nuclear level, such as nuclear lamin, chromatin, NPCs and other “nucleoskeletal” structures. Due to the high value of the lamin’s stiffness, it can be assumed that a great amount of force is transmitted to the other structures, thus affecting their geometry and mechanical properties (Jahed et al., 2014; García-González et al., 2016; Kirby and Lammerding, 2018). In particular, we hypothesize that the nuclear part of the NPC acts as a stretch-gated structure: if subjected to a force NPC can both alter the flux for the transported proteins and regulate the gene transcription. In the last 5 years we have correlated the mechanical stimuli with cell shape and nuclear geometry and we proposed a mechanism to explain how mechanical stimuli propagation affects the cell fate (Nava et al., 2012). So far there is no evidence that directly correlate the mechanical stimuli with NPCs activation. Nevertheless, several studies correlate the cell stretching with increased molecules flux in the nucleus. External mechanical stimuli applied either by AFM cantilever or stretch device, alter the nuclear/cytosolic YAP ratio as a consequence of the nucleus deformation (Elosegui-Artola et al., 2017; Ugolini et al., 2017). Same results were obtained in cells on high extracellular matrix rigidity where the nuclear import of YAP factor increases (Elosegui-Artola et al., 2017). As NPCs are the only gate between the nucleus and the cytoplasm, molecules flux alteration indirectly implies a pores regulation mechanism. To understand how the forces can be propagated, in this manuscript we provide an overview of NPC structure, function and connection with its environment. Finally, we analyze the pathologies correlated to the nuclear pore complex, highlighting how nuclear basket-related and lamina diseases appear to be linked.

## The Nuclear Pore Complex: Structure and Main Functions

**FIGURE 1:**
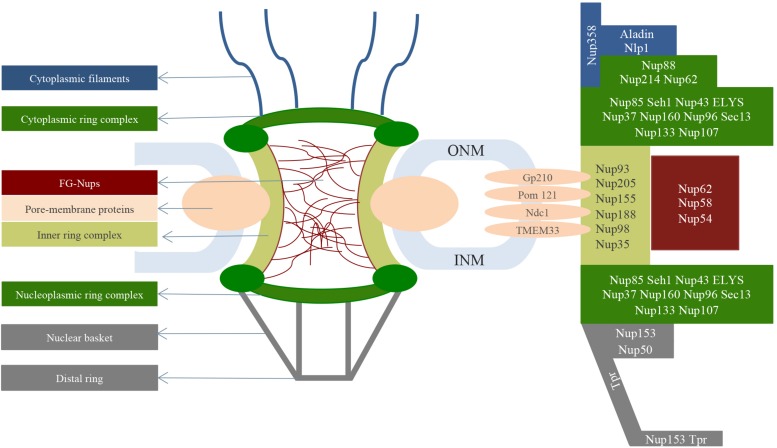
General structure of the NPC spanning the nuclear envelope (light blue). Starting from the cytoplasmic side, the cytoplasmic filaments (blue) bind the NPC central structure (green). The central scaffold is characterized by the cytoplasmic ring complex (dark green), inner ring (light green) and nucleoplasmic ring complex (dark green). FG-Nups (red) reside in the central channel where they allow the selective transport of molecules. In terms of the nucleoplasm, a structure called a nuclear basket (gray) anchors the central scaffold. On the right are shown the nucleoporins that make up the nuclear pore complex structures.

In eukaryotic cells, a coat protecting the genome material known as the nuclear envelope (NE) separates the nuclear compartment from the cytoplasmic compartment (light blue in Figure 1; Shahin, 2016). Nuclear pore complexes (NPCs) are large protein assemblies (110 MDa) that span the nuclear envelope with a density of around 2000–5000 NPCs/nucleus, varying according to cell size and activity (Miao and Schulten, 2009). Figure 1 summarizes the general structure of the NPC and its proteins. According to cryo-electron microscopy and tomography images, NPCs consist of an 8-fold symmetric central scaffold, eight cytoplasmic filaments, and eight nucleoplasmic filaments, resulting in a rotational symmetry (Lezon et al., 2009; Knockenhauer and Schwartz, 2016). The nucleoplasmic filaments co-join in a distal ring to form the so-called nuclear basket structure (Walther et al., 2001; Krull et al., 2004; Wälde and Kehlenbach, 2010; Moussavi-Baygi et al., 2011; Duheron and Fahrenkrog, 2015). The 8-fold rotational symmetry appears to maximize the bending stiffness of each of the eight NPC spokes, thus guaranteeing structural stability during the transport of large cargoes (Rowat et al., 2005). In addition, this configuration allows for the massive NPC to be built upon a comparatively small number of different nucleoporins (Rowat et al., 2005). Indeed, NPCs are assembled from only about 30 different proteins called nucleoporins (Nups), which reflect the octagonal symmetry of the NPC and occur in a copy number of eight or multiples of eight to give a total number of ∼1000 Nups in a single NPC (Wälde and Kehlenbach, 2010; Stanley et al., 2017). The Nups arrange in distinct sub-complexes joined to each other (Von Appen and Beck, 2016) and are typically categorized as (Figure 1):

- transmembrane Nups, also called pore-membrane proteins which form the so-called membrane ring;
- structural Nups (∼ 1/2 of all Nups) which form the NPC scaffold;
- channel Nups (∼1/3 of all Nups) also known as FG-Nups due to their richness in phenylalanine-glycine repeats;
- nuclear basket Nups;
- cytoplasmic filaments Nups.

### NPC Central Scaffold

The NPC central scaffold, is dominated by a α-solenoid domain, which guarantees flexibility and therefore conformational changes without breaking protein-protein interactions during nucleocytoplasmic transport (Von Appen and Beck, 2016). As represented in Figure 1, the scaffold pore structure is composed of three connected rings: the cytoplasmic and the nucleoplasmic ring complexes sandwich the inner ring complex (Stanley et al., 2017).

Cryo-electron tomography analysis indicates that both nucleoplasmic and cytoplasmic ring complexes (dark green in Figure 1) are characterized by the repetition of so-called Y subcomplexes (0.5–0.75 MDa) consisting of a stem base, which joins both the small and the large arm in a central hub element. Individual Y-shaped complexes are arranged head-to-tail into two antiparallel, ring-like octameric entities on both the cytoplasmic and nuclear face of the NPC (Duheron and Fahrenkrog, 2015; Eibauer et al., 2015). Both the nucleoplasmic and cytoplasmic ring complex structures consist of a total of 32 Y complexes assembled into two eight-membered, concentric and reticulated rings, which are stacked with a slight offset and differ only slightly in diameter (Von Appen et al., 2015; Hoelz et al., 2016; Knockenhauer and Schwartz, 2016).

The inner ring complex (∼90 nm in length, light green in Figure 1) spans the fused inner and outer nuclear membranes (light blue in Figure 1; Von Appen and Beck, 2016). It consists of eight similar “spokes” that stabilize the sharply bent nuclear envelope (Wälde and Kehlenbach, 2010). The inner ring complex harbors the central transport channel and additional peripheral channels of the NPC. Cryo-electron tomography showed that the central channel is an ordered structure forming a ring-like assembly called the central channel ring, which appears to be attached to the inner ring by a porous interface located ∼23 nm from the channel center (Eibauer et al., 2015). Anchored to the internal wall of the central channel (∼30–49 nm) are many unstructured and intrinsically disordered Nups containing hydrophobic sequences rich in phenylalanine-glycine repeats (such as FG, FxFG, GLFG) with ∼1 mM of concentration (Lemke, 2016; Stanley et al., 2017). Through their FG-repeats the central channel Nups (red in Figure 1) can interact, thus guaranteeing a compact morphology counteracted by repulsive effects due to charged amino acids of the same FG-repeats, which lead to protein extension (Stanley et al., 2017).

### Selective Transport Through the NPC Central Scaffold

Nuclear pore complexes are known to work as nucleocytoplasmic gateways that facilitate the selective transportation of thousands of molecules per second (∼100 MDa/s per NPC, transport process is completed in 5 ms) (Moussavi-Baygi et al., 2011; Chen et al., 2012; Knockenhauer and Schwartz, 2016; Stanley et al., 2017). They act in different ways depending on the molecules dimensions: while ions and small molecules diffuse passively through them, selected large cargoes are actively transported across the channel by signal-mediated mechanisms (Ball and Ullman, 2005; Alber et al., 2007; Miao and Schulten, 2009; Moussavi-Baygi et al., 2011; Hoelz et al., 2016; Jahed et al., 2016; Von Appen and Beck, 2016; Stanley et al., 2017). The FG-Nups located in the central channel appear to form a mesh with an effective size of 4–5 nm. This mesh allows the passive diffusion of molecules up to 40 kDa in size (9 nm) and facilitates the transport of those up to 25 MDa (39 nm), characterized by nuclear localization signals or nuclear export domains (Tu et al., 2013). The soluble nuclear transport receptor recognizes cargoes’ domains and shuttles the respective molecules between the nucleus and the cytosol (and vice versa), due to its affinity with the FG-Nups. To guarantee a high rate of transport, the interactions of the nuclear transport receptors with FG-Nups occur through very fast binding and unbinding kinetics (Moussavi-Baygi et al., 2011; Chen et al., 2012; Knockenhauer and Schwartz, 2016; Shahin, 2016; Stanley et al., 2017). Once the nuclear transport receptor-cargo complex is in the nucleus, RanGTP promotes the disassembly of the import complexes, freeing the cargo and allowing the nuclear transport receptors to diffuse back to the cytoplasm. In the cytoplasm, instead, RanGAP disassembles the export complexes.

**FIGURE 2:**
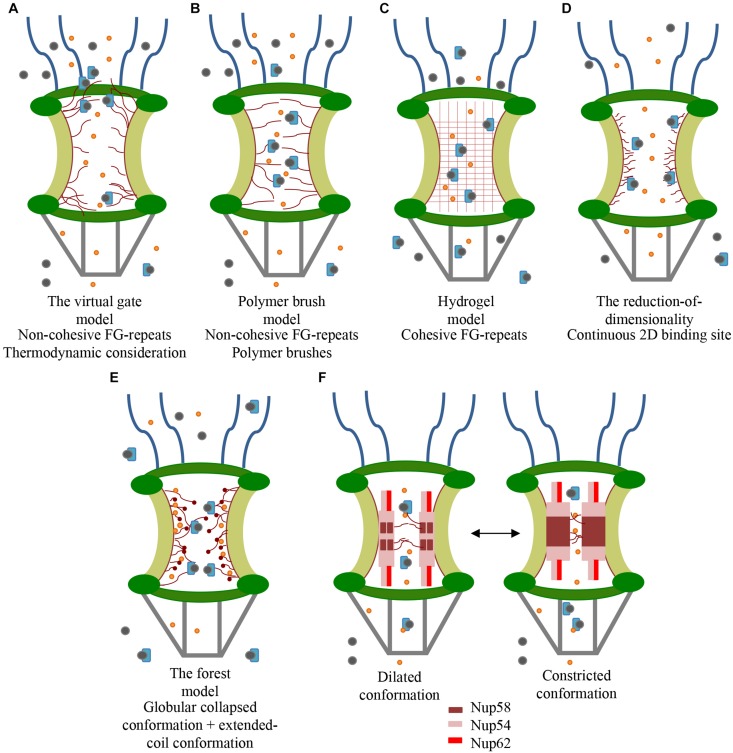
Proposed models for the FG-Nups distribution (red) in the central channel. Small proteins (orange) diffuse the channel passively. Only selected large cargoes (gray) can be transported across the channel by the nuclear transport receptor (light blue). **(A)** The virtual gate model envisions an entropic barrier, induced by FG-repeats dynamic configuration, which repeals unspecific cargoes. **(B)** The polymer brush model suggests bristles that reversibly collapse upon nuclear transport receptor binding. **(C)** The hydrogel model speculates that there is an homogeneous hydrogel mesh whose size excludes unspecific molecules passage. The high affinity of the nuclear transport receptor with the mesh would allow the passage of large selected molecules. **(D)** The reduction of dimensionality model supposes that a layer of FG-repeats lines the NPC interior forming 2D walk for and nuclear transport receptor. The axial channel allows for the passive diffusion of small molecules. **(E)** The forest model implicates both globular collapsed and extended-coil conformation for FG-Nups, which allow the passive diffusion of small molecules close to the scaffold (light green) and the active diffusion in the axial channel. **(F)** The central channel would alternate dilated and constricted conformation according to nuclear transport receptors’ concentration. While dilated conformation shows a single channel containing both Nup58 and Nup54, the constricted one consists of one homo-tetrameric module of Nup58 and two homo-tetrameric modules of Nup54.

Although the morphology of the FG-Nups in the central channel is essential for the selective transport, their structure is not well understood and several models have been proposed to explain the architecture of the NPC’s interior (Figure 2). According to the virtual gate model (Figure 2A), the unstructured non-cohesive FG-Nups form an entropic barrier at the periphery thanks to their thermal motion. While small molecules do not affect the FG-Nups domain motion and therefore can pass through the pore, large macromolecules restrict the FG-repeats motion with consequent increase in the required entropic price for the macromolecules transport. From the energy point of view, the large amount of the FG-repeats at the NPC’s periphery guarantees the entropic barrier lowering for nuclear transport receptor-cargo complexes; this facilitates the cargoes passage (Rout et al., 2003; Miao and Schulten, 2009; Moussavi-Baygi et al., 2011; Gamini et al., 2014; Ghavami et al., 2014). Consistent with the lack of FG-Nups stable interactions, experimental evidence showed that a layer of FxFG-repeats of Nup153 results entropically repulsive; moreover, electron microscopy analysis showed topological flexibility of FG-repeats (Terry and Wente, 2009). Nevertheless, other evidences reported *in vitro* hydrogel formation from FG-Nups in contrast with the existence of a non-cohesive barrier (Frey et al., 2006). In addition, the reduction of up to half of the FG-repeats mass seems do not affect the NPCs permeability (Strawn et al., 2004). This observation is in contrast with the necessity of high FG-repeats concentration required for the strong entropic barrier formation (Terry and Wente, 2009). In addition, the 3D spatial-density map of Importin β1 and FG-repeats interactions suggests higher entropic barrier in the central pore and lower at the periphery (Yang, 2011). Quite similar to the previous one, the polymer brush model (Figure 2B) proposes that FG-Nups form extended brush-like polymers with bristles that reversibly collapse upon nuclear transport receptor. The nuclear transport receptors are indeed supposed to push aside the non-cohesive FG-Nups providing a kinetic advantage for the cargo passage (Yang, 2011). Repetitive binding and unbinding events between nuclear transport receptor and FG-repeats would guarantee the transport of the cargo (Miao and Schulten, 2010; Moussavi-Baygi et al., 2011; Gamini et al., 2014). Consistent with this theory, AFM analysis on surface-tethered Nup153 showed FG-repeats reversible collapse in presence of the Importin β1 (Lim et al., 2007). Further studies on Nup62 revealed the nucleoporin collapse as function of both transport receptors concentration and the FG-Nups grafting distance (Ghavami et al., 2014). The hydrogel model (Figure 2C) suggests that the hydrophobic FG-repeats interact in the central channel forming a sieve-like hydrogel meshwork. The latter is reversibly dissolved by nuclear transport receptors allowing the cargoes passage through the channel (Ribbeck and Görlich, 2002; Miao and Schulten, 2009; Gamini et al., 2014). Consistent with this theory, *in vitro* tests showed that, at least in altered pH condition, the hydrophobic interaction between aromatic rings of the Nsp1 (Saccharomyces cerevisiae nucleoporin) form an elastic hydrogel. Same result has been achieved by using a composed mix of the Saccharomyces cerevisiae nucleoporins Nup49, Nup57, and Nsp1 (Frey et al., 2006; Frey and Görlich, 2009). Although the obtained hydrogel-like structure mimics the selective properties of the NPC in terms of time required for the molecules passive diffusion, there is no evidence about hydrogel formation in the physiological environment (Ghavami et al., 2014). In this context, a computational Monte Carlo study demonstrated the possibility of gel formation, but at a concentration level (42 mg/mL) far from experimental gel transition one (8–10 mg/mL) (Ghavami et al., 2014). According to this model, NPCs lacking Nup98 (nucleoporin rich in cohesive GxFG-repeats) do not provide molecules barrier anymore and affects the molecular import. The barrier could be restored by Nup98 addition, while introducing non-cohesive FG-repeats cannot reinstate a proper barrier (Hülsmann et al., 2012; Powers and Forbes, 2012). These results suggest that the pore selectivity depends on both inter-FG-Nups and nuclear transport receptor-FG-Nups interactions (Hülsmann et al., 2012). Instead, the reduction of dimensionality model (Figure 2D) suggests that a layer of FG-repeats lines the NPC interior and that the nuclear transport receptor slides on the surface of FG-repeats according to a 2D random walk (Miao and Schulten, 2009; Moussavi-Baygi et al., 2011; Gamini et al., 2014). Accordingly, studies on the topology of the NPC suggested that FG-Nups form a hydrophobic layer that cover the central channel (Ghavami et al., 2014). In addition, single particle tracking experiments and SPEED microscopy analysis showed the molecules passive diffusion through the central region while the active transport is closer to the scaffold (Yang, 2011; Ghavami et al., 2014). In contrast with these data, the forest model (Figure 2E) predicts that the macromolecules would pass through the center of the channel and that the passive transport occurs close to the NPC scaffold. Indeed, this model features two separate zones: while the interior of the NPC is assumed as a hydrogel, the area close to the scaffold is presented as a brush-like mesh. According to this model, the Nups are classified as “shrubs,” which form cohesive domains with collapsed-coil and low-charge content, and “trees,” with a collapsed-coil on top of an extended coil that form high-charge structure rather than cohesive domains (Moussavi-Baygi et al., 2011; Gamini et al., 2014). The identification of about eight symmetrical pores around the central channel supports this model. Indeed, these pores seem to be an alternative pathway for the passive transport of small molecules and ions (Bootman et al., 2009; Koh and Blobel, 2015). Moreover, the analysis of the FG-repeats hydro-dynamic properties showed the presence of five distinct domains: collapsed-coil cohesive domain, collapsed-coil non-cohesive domain, extended-coil cohesive domain, folded domain and NPC anchor domain. In light of that, the collapsed-coil domains would represent the “shrubs” and the extended one would form the “trees.”

More than one model can exist in parallel for FG-Nups, and different states may also be in dynamic equilibrium (Lemke, 2016). However, all of these models seems to be in contrast with numerous electron microscopic studies, which demonstrate the dynamic nature of the central channel (Au and Panté, 2012; Koh and Blobel, 2015). Indeed, since 1990, cryo-electron microscopy revealed several forms of the central channel spanning from a constricted to a dilated form (Hansen and Ingber, 1992). In light of that, another transport model is proposed based on the role of two channel Nups (Nup54 and Nup58), which can reversibly form distinct homo- and hetero-oligomers (Figure 2F). These oligomers are assumed to be the building modules of “midplane rings” alternating between constricted and dilated conformations in the central channel. The dilated conformation (Figure 2F, left) shows a channel of ∼40 nm in diameter containing eight copies of the hetero-dodecameric module, consisting of four Nup58 and eight Nup54. The constricted configuration (Figure 2F, right) is instead characterized by one homo-tetrameric module of Nup58 and two homo-tetrameric modules of Nup54 which form a channel of ∼20 nm in diameter. The equilibrium between the two conformations can be regulated by the nuclear transport receptor’s concentration: a high concentration corresponds to dilated conformation. In both the conformations, Nup54 interacts with Nup62 (ratio 1:2) forming “finger-shaped” triple helices (32 on both the nucleoplasmic and cytoplasmic side) before splitting into unstructured appendages characterized by FG-repeats (Solmaz et al., 2013; Koh and Blobel, 2015). Consistent with this model, electron micrographs of NPC cross-sections from Xenopus oocytes support a model in which the central channel is totally emptied both in length and in width to accommodate the macromolecules (Au and Panté, 2012).

### The Cytoplasmic Filaments

On the cytoplasmic face, the NPC is characterized by eight short filaments (30–35 nm in length) with free distal ends (blue in Figure 1), whose main constituent is the nucleoporin Nup358 (Krull et al., 2004). Nup358 is anchored by its N-terminus to the cytoplasmic ring complex’s Nup133 and Nup96. Several other sub-complexes essential for the mRNA transport and the nuclear transport receptor-cargo complex assembly and disassembly, bind to the scaffold on the cytoplasmic side (Von Appen and Beck, 2016).

### The Nucleoplasmic Filaments: The Nuclear Basket

The nucleoplasmic ring complex is capped by a fibrous structure called the nuclear basket (gray in Figure 1), consisting of eight thin fibrils (∼40–75 nm in length) appended to the nucleoplasmic ring complex and interconnected at their distal ends where they form the distal ring (Krull et al., 2004). The nuclear basket contains nucleoporin Nup153, Tpr and Nup50. Nup153 binds Tpr via residues 228–439 and Nup50 via residues 337–611, while Nup50 does not have binding sites for Tpr (Duheron et al., 2014).

Tpr has been proposed as the central basket architectural element, linked to the central scaffold via direct binding to the Nup153 (Krull et al., 2004; Duheron et al., 2014). In this context, immuno-electron microscopy studies of domain-specific antibodies suggest that the basket fibers may be formed by Tpr dimers folded back onto themselves with both N- and C-termini located at the distal ring (Duheron et al., 2014). The folding of a Tpr dimer can occur at the “NPC binding domain,” which binds the Nup153 N-terminal domain. The intradimer interactions would then take place along the first two thirds of Tpr’s rod domain; the last third, together with the other Tprs’ C-terminal tail domains, constitutes the distal ring (Krull et al., 2004). Tpr primary sequence indicates the presence of two distinct domains in the protein: (i) a coiled-coil alpha-helix in the region between residues 50 and 1630 (there are several short (<50 residues) segments interspersed in this region with low coiled-coil potential) and (ii) a 700-residues hydrophilic domain enriched with acid residues, serine and threonine (Byrd et al., 1994). Residues 436–606 appear to be required, and are sufficient to localize Tpr to NPCs (Duheron et al., 2014).

Another constituent of the NPC’s nuclear basket is Nup153 (1475 amino acids) composed of three main domains: (i) the N-terminal domain spanning about 600 amino acids (Duheron et al., 2014), which consists of a nuclear localization signal, a nuclear envelope targeting cassette, an NPC association region and a RNA binding domain (Ball and Ullman, 2005; Al-Haboubi et al., 2011; Duheron and Fahrenkrog, 2015); (ii) a central domain spanning about 250 amino acids consisting of four or five zinc fingers, which tie Nup153 to the distal ring (Duheron et al., 2014); (iii) a C-terminal domain consisting of around 600 amino acids containing ∼30 FxFG-repeats (Walther et al., 2001). The N-terminal domain is predominantly detected closer to the membrane, the zinc-finger domain is found at the distal ring, whereas the Nup153’s FG-repeats is detected all over the nuclear basket and occasionally on the cytoplasmic side (Ball and Ullman, 2005; Duheron and Fahrenkrog, 2015). The nature of Nup153 localization is not fully elucidated yet and several scenarios have been proposed to explain how a mobile Nup could tether a stable component such as Tpr. One hypothesis suggests that only a subset of Nup153 is required at the NPC to fulfill its anchoring role. This scenario indeed shows two distinct populations of Nup153 at the NPC, one in dynamic association and one stably integrated (Walther et al., 2001; Ball and Ullman, 2005). Another theory instead proposes that Nup153 delivers Tpr to the NPC, but does not remain stably engaged once Tpr is incorporated via other interactions. It is known that the nucleoporin Nup153 is involved in multiple nuclear processes, such as nuclear protein import, RNA export, nuclear assembly/disassembly, mitosis and cell cycle progression (Al-Haboubi et al., 2011). Furthermore, Nup153 affects cellular processes due to its direct interactions with transcription factors, signaling molecules, membrane remodeling proteins and SUMO specific protease, and plays an essential role in maintaining nucleoskeleton/cytoskeleton architecture, cell migration and gene regulation (Zhou and Panté, 2010; Al-Haboubi et al., 2011; Duheron and Fahrenkrog, 2015). It is of interest that NPCs lacking Nup153 proteins are more mobile within the NE with consequent NPCs clustering and redistribution (Walther et al., 2001; Goldberg, 2017). In addition, NPCs lacking Nup153 show altered nuclear lamina, cytoskeleton and SUN1 organization and result in the loss of several Nups such as Nup93, Nup98, and Tpr (Walther et al., 2001; Zhou and Panté, 2010; Duheron and Fahrenkrog, 2015). Despite the supposed role of Nup153 in NPCs anchoring, it has been assumed that NPCs maintain their localization in the nuclear envelope mainly due to four different Nups (pore-membrane proteins), which form the so-called membrane ring (light pink).

## Structural Biology of the Connection Between the NPC and Its Environment

Nuclear pore complexes are fixed in the nuclear envelope by tethering to their environment. In addition to the aforementioned pore-membrane proteins, there are several other connections between NPCs and their surroundings. NPCs mainly connect with the lamina, the LINC complex and the chromatin.

### NPC Connection With the Lamina

The nuclear lamina is a structure close to the inner nuclear membrane, composed of lamin and lamin-associated proteins, which form a network proving structural support to the cell nucleus. Blot overlay assay revealed lamin-binding domains in Nup153 at both N- and C-termini, which appear to link the Ig-fold domains of both lamins A and B (Al-Haboubi et al., 2011). Supporting this, Nup153-depleted cells showed altered nuclear lamina organization. In addition, mutations in the lamin Ig-fold domain correspond to altered Nup153 localization with potential effect on lamin-associated diseases (Gruenbaum et al., 2005; Zhou and Panté, 2010; Al-Haboubi et al., 2011).

### NPC Connection With LINC Complex

**FIGURE 3:**
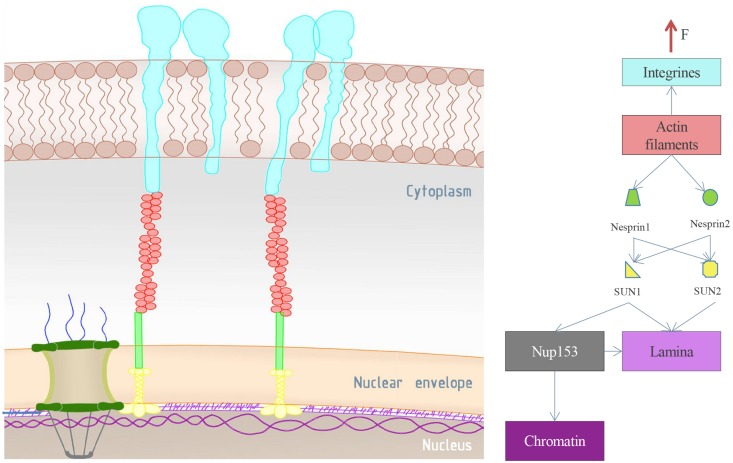
Mechanical connections of the NPC. On the left are the NPC and its environment. Integrins are represented in light blue. They span the outer cell membrane and transmit the external stimuli to the interior of the cell. Integrins are mainly connected to actin filaments (red), which in turn are connected to the LINC complex (the green and the yellow segment represents Nesprin and SUN proteins, respectively). The LINC complex can link the lamina (light purple) or the NPC via Nup153 connection (gray). In addition, the nucleoporin Nup153 may be connected to both the lamina and the chromatin (dark purple), as schematically reported in the image on the right side. The reported scheme represents a possible pathway of external forces transmitted from the environment to the NPC basket (Nup153).

The LINC complex is a protein bridge across the nuclear envelope mediated by SUN (Sad1-UNC-84 homology) and Nesprin proteins (Klarsicht, ANC-1, and Syne homology) localized at the outer and inner nuclear membranes, respectively (Starr and Fridolfsson, 2010; Sosa et al., 2012; Gay and Foiani, 2015; Goldberg, 2017). While the Nesprin cytoplasmic domain associates with cytoskeleton filaments, the C-terminus is instead characterized by a short KASH peptide of around 30 residues, which interacts with SUN protein (Sosa et al., 2012). There are at least four human Nesprins, which differ in their ability to bind different cytoskeleton filaments and SUN proteins: Nesprin1 and Nesprin2 bind actin filaments at their actin binding domains and SUN1 and SUN2 proteins at the luminal KASH peptides; Nesprin3 binds to intermediate filaments via Plectin proteins; and Nesprin4 links to microtubules through Kinesin1 motor protein (Sosa et al., 2012; Jahed et al., 2014; Nie et al., 2016; Goldberg, 2017; Figure 3). A structural and biochemical characterization of the SUN-KASH domains complex shows three KASH peptides binding a SUN trimer (Sosa et al., 2012; Zhou et al., 2012; Nie et al., 2016). The SUN proteins are transmembrane proteins crossing the inner nuclear membrane and interacting with the nuclear lamina, the chromatin and other “nucleoskeletal” structures at their N-termini (Jahed et al., 2016; Goldberg, 2017), and with the KASH domain at their C-terminus. SUN1 and SUN2 are more widely expressed SUN proteins in various cell types, and seven isoforms have been identified (Jahed et al., 2014). Pull-down experiments suggest that SUN1 may interact with Nup153 (Figure 3), and that both the N- and the C-termini are involved in this binding. This evidence is supported by immunofluorescence microscopy, which reveals that SUN1 colocalizes with Nup153 (Li and Noegel, 2015). In addition, some studies have revealed the NPC-altered localization in SUN1-depleted cells and SUN1-altered position in Nup153-depleted cells (Liu et al., 2007; Zhou and Panté, 2010; Li and Noegel, 2015; Goldberg, 2017; Weiyi et al., 2017). These elements can provide an indirect coupling between the Nup153 of the NPC basket and the cytoskeleton elements, resulting in NPC exposure to cytoskeletal forces (Jahed et al., 2016). The physical connection between Nup153 and the cytoskeleton may play a role in avoiding NPC clustering, defining their nuclear positioning and anchorage, affecting cytoskeletal organization and imposing nuclear sizing and architecture (Zwerger et al., 2011). In addition to Nup153, studies have shown that the Nup358 N-terminus interacts with interphase microtubules via motor proteins such as dyneins and kinesins (Joseph and Dasso, 2008; Jahed et al., 2016; Goldberg, 2017). Finally, electron microscopy and cryo-electron tomography analyses revealed that vimentin, an intermediate filaments, could be associated with cytoplasmic ring complex, but there are no biochemical or functional data confirming such a link (Goldberg, 2017).

### NPC Connection With Chromatin

In addition to the lamin and the LINC binding domains, NPCs are characterized by chromatin binding regions. Although the nuclear periphery was previously associated with transcription repression, recent studies on the chromatin demonstrate the coexistence of repressive and active domains, close to the NE and the NPC, respectively (Texari and Stutz, 2015). NPC substructures such as nuclear basket Nups, scaffold Nups and central channel Nups associate with numerous actively transcribing genes (Ptak et al., 2014). From a ChIP-on-chip analysis in yeast, it was found that several activated genes re-localize at the nuclear periphery due to direct or indirect affection induced by basket nucleoporins mutations (Texari and Stutz, 2015). Again, nucleoporin Nup153 appears to play a role and it can be considered as a chromatin-binding protein: it binds to 25% of the genome in domains termed nucleoporin-associated regions, which demarcate the regions of open chromatin and transcriptional activity. The widespread transcriptional changes revealed in Nup153-depleted cells appear to confirm the role of Nup153 in open chromatin environment formation and gene expression control (Vaquerizas et al., 2010; Duheron et al., 2014). Although the gene localization in the NPC is clearly correlated with the transcription, the underlying mechanism has not been yet identified. One hypothesis suggests the implication of sumoylation/de-sumoylation dynamics in fine-tuned gene expression (Texari and Stutz, 2015). Other studies have demonstrated that only the Nups in the nucleoplasm, away from the NPC, stimulate developmental and cell-cycle genes expression (Capelson et al., 2010; Kalverda et al., 2010). Thus, although it has been shown that NPCs may be involved in gene regulation, their role remains elusive (Capelson et al., 2010).

## Mechanosensing Response at the Nuclear Envelope: From External Stimuli to Gene Regulation

**FIGURE 4:**
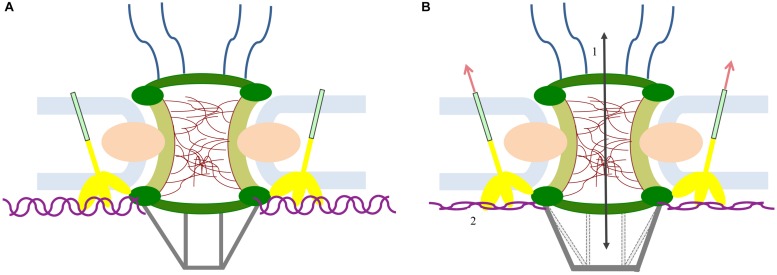
Hypothesis of the mechanism of NPC stretch activation. **(A)** NPC rest condition. **(B)** External forces act on SUN1 (yellow), which induces the basket opening. The basket opening may influence the NPC permeability with consequent flux alteration (1). Moreover, the basket stretching may rearrange the architecture of the DNA genes directly connected to it (purple) (2).

Cells anchor the extracellular matrix via focal adhesions, which transmit external mechanical stimuli to cytoskeleton filaments up to the LINC complex (Figure 3). External stimuli transmission induces distortion in the nucleus and modulates the expression of mechanoresponsive genes via several proposed mechanisms, which could alter chromatin organization, transcription and other cellular processes (Ribbeck and Görlich, 2002; Jean et al., 2004; Wang et al., 2009). Some evidence showed that transmitted forces may move inactive heterochromatic DNA from the nuclear periphery to the interior of the nucleus, facilitating transcription factor accessibility and activity (Kirby and Lammerding, 2018). Other experiments show that the mechanical load of 5 pN of force induces chromatin decondensation within less than 30 s, which results in a transcription increase in the stretched region. Interestingly, the long-term effect of a persistent mechanical load reflects a mechanoadaptive process with gene silencing effects, which can act as a negative feedback mechanism (Miroshnikova et al., 2017; Kirby and Lammerding, 2018). In addition to chromatin alteration, nuclear deformation can cause local crowding and the exclusion of soluble factors associated with nuclear processes alteration; the exclusion of transcriptional regulators can indeed affect transcriptional activity (Kirby and Lammerding, 2018). Due to the NPCs mechanical linkages with the NE, the LINC complex and the lamina, it was supposed that mechanosensed nuclear deformations could also affect the structure of the pores (García-González et al., 2016). During cell-extracellular matrix adhesion, the cytoskeleton rearrangement does in fact transmit tensile forces to the Nesprin, allowing the physical force propagation till the SUN proteins, which in turn will discharge the force onto structures they are linked with. In particular, SUN1 can mainly transmit these mechanical forces either to the lamina or to the NPC structure, consequently affecting NPC permeability (Jahed et al., 2014; Nava et al., 2016; Kirby and Lammerding, 2018). In 1990, cryo-electron microscopy studies reveled that spread cells allow higher molecular transport compared to the nuclei of the cells in roundish configurations (Hansen and Ingber, 1992). Several data have been so far collected and more recent studies showed that the nuclear stretch, caused by different mechanical stimuli, induces increase in the nuclear import of YAP factor (Elosegui-Artola et al., 2017; Ugolini et al., 2017). To explain the obtained data, the authors suggested that high value of substrate rigidity induces a force transmission to the nucleus with consequent pores opening and faster molecular import in the cytoplasmic side (Elosegui-Artola et al., 2017). According to this theory, current models propose that the NPC central channel can constrict or dilate, changing their mechanical impedance to the molecular transport (Solmaz et al., 2013; Chug et al., 2015). Nevertheless, from recent tomographic 3D reconstruction of the nuclear envelope it has been demonstrated that the spread and the roundish configurations show similar pore areas values (García-González et al., 2018). These data seem to exclude the central channel as main actor in molecules transport regulation. Indeed, the authors suggested that changes in the nuclear pore complex permeability could be due to nuclear basket rearrangement (García-González et al., 2018). Based on these evidences and due to the interaction of SUN1 with Nup153 (Li and Noegel, 2015), we developed a new model based on the mechanoactivation of NPC, where the nuclear basket portion can act as a stretch-gated structure. External stimuli will be then transmitted by SUN1 to Nup153, which will stretch Tpr basket protein, inducing a reorganization of the basket structure. Changes in basket conformation would induce higher or lower flux for the transported proteins depending on the basket opening or closing, respectively (Figure 4, effect 1). The proposed model is consistent with the evidence that the LINC complex blocking causes impaired YAP translocation and therefore pores alteration (Elosegui-Artola et al., 2017). In addition, since 1999 it is known that the distal ring of the basket may adopt structural changes ranging from constricted and dilated conformation even if the only trigger event verified so far is the calcium concentration (Stoffler et al., 1999). Moreover, the association of the basket with active genes implies transcription regulation according to basket size changes (Figure 4, effect 2) (Wang et al., 2009; Ptak et al., 2014). Indeed, the Nup153 stretching may induce changes in the condensation grade of the chromatin domain it is connected to. In line with this theory, other studies indicate that Nup153-binding induces a high-level of transcription activity, probably promoted by both the open chromatin status and the higher nuclear import in spread cells compared to roundish cells (Vaquerizas et al., 2010; García-González et al., 2018). Eventually, the correct mechanical activation of the nuclear basket may be influenced by the lamina architecture. Indeed, the SUN1 force distribution between the lamina and the Nup153 may play a role in the cells’ physio-pathological behavior. Alteration of the lamina structure would induce a pathological force transmission on the Nup153, affecting the correct behavior of the nuclear basket. Thus, it is clear how laminopathies may also be closely related to basket dysfunctions.

## Diseases Correlated to Nups Mutation

Several diseases correlate with most of the connections revealed between NPCs and neighboring structures such as the LINC complexes and the lamina, although the causal link is not known. In Tables 1, 2 we summarize the pathologies correlated to NPC Nups, to the LINC complex and to the lamins. In addition to the related proteins, for each pathology we report the main observed aberration, such as gene fusion (“GF”), transmembrane flux variation (“F”), depletion of protein (“D”), overexpression of protein (“O”), gene mutation (“M”), protein improper localization (“L”) or other (“X”). The data in the table suggest that most of the structural Nups correlate with cardiological disease, types of cancer and other pathologies such as amyotrophic lateral sclerosis. However, the correlation among nuclear basket Nups, the LINC complex and the lamins in laminopathies is evident. For example, mutations in the LMNA gene, the encoding gene for lamins A and C, are implicated in Emery-Dreifuss muscular dystrophy, limb girdle muscular dystrophy, dilated cardiomyopathy, Dunningan-type familial partial lipodystrophy, and Hutchinson-Gilford progeria syndrome (Philip and Dahl, 2008; Wang et al., 2009). SUN1 interacts directly with lamin A and therefore its role in laminopathies was investigated. Surprisingly, knocking down SUN1 in Hutchinson-Gilford progeria syndrome cells cultured *in vitro* prevented heterochromatin loss and accelerated senescence; in contrast, an over-accumulation of the SUN1 protein in the NE and the Golgi apparatus triggers nuclear envelope rupture (Chen et al., 2012; Chi et al., 2012; Liu et al., 2012). Although several studies address this, the molecular mechanism underlying these diseases remains unclear. In any case, the abnormally shaped nuclei and the changes in chromatin organization revealed in pathological cells suggest structural or gene regulation origins (Muchir and Worman, 2004; Gruenbaum et al., 2005; Wang et al., 2009; Zwerger et al., 2011). The structural hypothesis suggests that the functional loss of lamins A and C could increase nuclear fragility and cell death in mechanically stressed tissue such as muscle (Gruenbaum et al., 2005; Wang et al., 2009). Thus, most of the laminopathies-affected tissues are found to be under mechanical stress in the body, highlighting the mechanotransduction role in laminopathies (Philip and Dahl, 2008). For example, skeletal muscle fibers from Emery-Dreifuss muscular dystrophy patients contain fragmented nuclei (Zwerger et al., 2011). Although studies have confirmed that fibroblasts of laminopathy patients show reduced resistance to mechanical stress, Hutchinson-Gilford progeria syndrome is caused by the increased presence of wild-type and mutant lamin A, resulting in stiffer, less compliant nuclei with changes in their interior chromatin organization, loss of heterochromatin condensation, and accumulation of DNA damage (Philip and Dahl, 2008; Wang et al., 2009). In this context, the hypothesis concerning gene regulation attempts to explain how Hutchinson-Gilford progeria syndrome affects load-bearing tissues, proposing that the NE interaction with chromatin regulates tissue-specific gene expression and that mutation in lamins alters this regulation (Muchir and Worman, 2004). In addition, fibroblasts derived from lamina-null mouse embryos showed an impaired mechanically activated gene transcription (Crisp et al., 2006). Structural and gene regulation hypotheses are not mutually exclusive and could be interrelated by nuclear mechanotransduction (Wang et al., 2009). For instance, Hutchinson-Gilford progeria syndrome nuclei do induce an altered shear stress response, while they also show changes in gene expression (Philip and Dahl, 2008; Wang et al., 2009). Thus, changes in nuclear structure and function could contribute both to increased cellular sensitivity to mechanical strain and to altered transcriptional regulation (Wang et al., 2009). Within this context, our proposed nuclear basket stretch activation model can contribute to explain the mechanisms for the laminopathies’ etiology. Laminopathic diseases such as familial partial lipodystrophy of the Dunningan type and Emery-Dreifuss muscular dystrophy indeed show lamin A mutation in the Ig-fold domain, where the lamins are assumed to be connected to the basket Nup153. This suggests that Nup153 has a role in the etiology of laminopathies (Al-Haboubi et al., 2011; Jahed et al., 2016). In addition, it has been noted that mutations in the LMNA gene correlate with a decrease in Nup153 at the nuclear envelope (Duheron and Fahrenkrog, 2015). Alterations in lamin A and C imply an impaired mechanoresistance of the nuclear lamina with consequent alteration in mechanotransduction from the LINC complex (Philip and Dahl, 2008; Miroshnikova et al., 2017). Here we suggest that a defective mechanotransduction can induce an alteration in basket opening, causing both impairment in the transport of molecules through the NPC and changes in chromatin organization. Supporting this hypothesis, studies have revealed that lamin A alteration has an impact on both the localization and distribution of NPC proteins implicated in molecular transport and on the decreased import pathway (Busch et al., 2009). Again, other evidence shows that the altered protein import is associated with Hutchinson-Gilford progeria syndrome, restrictive dermopathy and aging (Busch et al., 2009). Altered mechanotransduction may also be due to the destruction of LINC complexes, which could be the cause of other laminopathies such as Emery-Dreifuss muscular dystrophy and dilated cardiomyopathy, and neural disorders such as lissencephalites (Stewart-Hutchinson et al., 2008; Sosa et al., 2012; Nie et al., 2016). LINC destruction can cause impaired force transmission to the nuclear basket, and basket molecular structures, such as Nup153 and Tpr, are likely to be the primary cause of laminopathies. In addition to laminopathies, deregulated lamin expression has been observed in several cancers (Matsumoto et al., 2015). Loss of lamin A and C expression has been revealed in colon cancer, breast cancer, small cell lung cancer, leukaemias and lymphomas. In contrast, the overexpression of lamin A and C has been reported in skin cancer, colorectal cancer, and prostate cancer (Matsumoto et al., 2015). There is also evidence of a correlation between a loss of SUN1 expression and breast cancer initiation and/or progression. In addition to the LINC complex, the loss of Nups expression also appears to be correlated with cancer (Duheron and Fahrenkrog, 2015). Although the precise functions of reduced LINC complex remain elusive, it has been suggested that the loss of LINC complex (SUN1, SUN2, Nesprin2) might alter nuclear structure and mechanical properties, affecting genome integrity, proliferation, and cell migration with consequences for cancer progression, and may induce impairment in DNA repair, which plays a role in tumor initiation (Matsumoto et al., 2015).

**TABLE 1:** Published studies showing correlation between diseases and Nups, lamins, and the LINC complex.

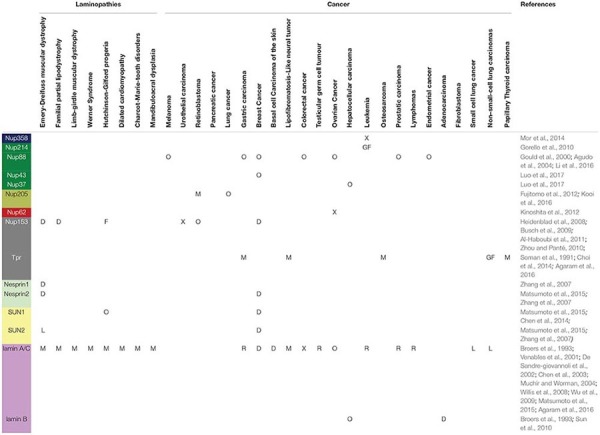

*In the table the diseases are classified as laminopathies and cancer disease. Different causes are indicated: gene fusion (“GF”), transmembrane flux variation (“F”), depletion of protein (“D”), overexpression of protein (“O”), gene mutation (“M”), protein improper localization (“L”), other (“X”).*

**TABLE 2:** Published studies showing correlation between diseases and Nups, lamins, and the LINC complex.

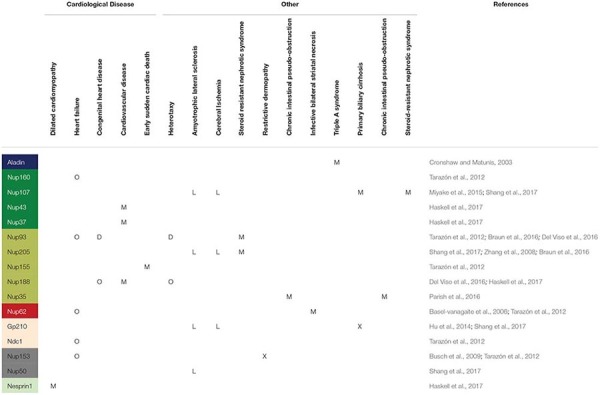

*In the table the diseases are classified as cardiological disease and other. Different causes are indicated: gene fusion (“GF”), transmembrane flux variation (“F”), depletion of protein (“D”), overexpression of protein (“O”), gene mutation (“M”), protein improper localization (“L”), other (“X”).*

## Conclusion

Several studies investigated nuclear involvement in the cell mechanotransduction and cell fate but only in recent years the research has been trying to correlate this mechanism with the architecture and function of the NPCs. Several aspects remain so far elusive. Based on the literature, we summarized the main experimental evidence and proposed models showing a mechanosensing role of the NPC and its relationship with several pathologies. We hypothesize that the nuclear basket has a key role as the primary regulator of NPC transport, by means of a mechanoactivation mechanism involving SUN1 and Nup153 proteins. Our group is further exploring the possibility of verifying the behavior of the nuclear basket as a stretch-gated structure, using biophysical and computational methods. Specifically, we are investigating the molecular arrangement of the Nup153-SUN1 complex through molecular dynamic studies based on homology modeling predictions, and with X-ray crystallography techniques. At a broader level, we plan to develop a model to evaluate external force transmission and its effects on the nuclear pore complex structure. This engineering approach should lead to a more quantitative understanding of basket structure alteration and its potential effect on transcription and disease.

## Author Contributions

All authors listed have made a substantial, direct and intellectual contribution to the work, and approved it for publication.

## Conflict of Interest Statement

The authors declare that the research was conducted in the absence of any commercial or financial relationships that could be construed as a potential conflict of interest.
